# Viscoelasticity analysis of coarse-grained cytoskeletal simulations with Cytosim and Cytocalc

**DOI:** 10.1016/j.bpj.2026.02.026

**Published:** 2026-03-04

**Authors:** Krishna Iyer V.S., Komal Bhattacharyya, Raffaele Mendozza, Peter K. Sollich, Stefan Klumpp, Yoav G. Pollack

**Affiliations:** 1 University of Göttingen, Institute for the Dynamics of Complex Systems, 37077 Göttingen, Germany; 2 Indian Institute of Science Education and Research, Department of Physics, Pune, India; 3 University of Göttingen, Institute for Theoretical Physics, 37077 Göttingen, Germany; 4 King’s College London, Department of Mathematics, Strand, London WC2R 2LS, UK

## Abstract

Computational modeling has emerged as a powerful approach to studying cytoskeletal dynamics. The simulation software Cytosim provides intuitive yet flexible simulations of filament polymerization, cross-linking, and motor activity. Here, we present Cytocalc, a lightweight Python toolkit designed to streamline and standardize the analysis of Cytosim simulation output, supporting studies of biological functionality and physical properties of cytoskeletal systems. After introducing Cytocalc and validating it, we use it to establish a new workflow for quantifying network viscoelasticity from Cytosim simulations. Specifically, we determine the complex shear modulus of cross-linked networks and quantify how the storage modulus increases with cross-linker density. The cross-linker dependence of the network’s elasticity exhibits two regimes, a scaling regime consistent with elasticity arising from the suppression of thermal bending fluctuations of filaments as well as a much weaker dependence at high cross-linker concentration.

## Significance

The mechanical behavior of cytoskeletal networks underlies key cellular processes such as shape control, migration, and force transmission. While Cytosim simulations are widely used to study filament dynamics, quantitative analysis often relies on custom, ad hoc scripts. Cytocalc provides an open, standardized toolkit for analyzing Cytosim output and enables, for the first time to our knowledge, systematic rheology studies within the framework. Using Cytocalc, we quantify how network elasticity depends on cross-linker density and identify distinct regimes governed by filament bending and connectivity changes. By reducing technical barriers and promoting reproducible workflows, Cytocalc enhances numerical studies of cytoskeletal mechanics, links microscale dynamics to emergent material properties, and lays the foundation for future active rheology investigations.

## Introduction

The cytoskeleton is a dynamic filamentous network that supports essential processes such as cell division, migration, and morphogenesis (1,2,3). Its capacity to reorganize and generate forces in response to mechanical cues emerges from the collective behavior of actin filaments, microtubules, motor proteins, and cross-linkers (4,5). Key findings on component dynamics, such as burst-like actin severing by cofilin (6) and rapid microtubule catastrophe-rescue cycles (7) offer important clues about the link between molecular events and cell-scale mechanics. In particular, the cytoskeleton has been shown to underlie the cell’s viscoelastic properties (8), including the characteristic power law response of living cells (9,10). Understanding these rheological properties is therefore central to connecting microscopic interactions with emergent mechanical behavior.

Several theoretical models ranging from polymer theory to mesoscopic models of disordered systems have been proposed to rationalize the physical mechanism leading to the power law rheology exhibited by the cytoskeleton (11,12). Numerically, such responses have been investigated using simulations of 2D or 3D lattice-based networks (13), simulation of slender fibers in flow using integral-based spectral method with either permanent cross-linkers (14) or transient cross-linkers (15), or systems with semiflexible filaments represented as bead-spring chains either with (16) or without (17,18) permanent cross-linkers. These approaches can capture some dynamic features of the cytoskeleton (19) including viscosity divergence at the isotropic-nematic transition (20) and activity-induced anomalous elasticity (21). However, motor-driven activity in these models is typically implemented via simplified force laws, whereas in simulation platforms that explicitly incorporate motor binding-unbinding, stepping, and force-velocity relations, such viscoelasticity investigations are mostly lacking.

Cytosim (22,23) is an agent-based simulation platform that overcomes these limitations, enabling modeling of various active cytoskeletal components. It has been applied to a wide range of cytoskeleton-related biophysical systems and processes including actomyosin contractility (24), centrosome positioning (25), and actin dynamics in endocytosis (26). Its open-source code, extensible object model, and efficient Brownian dynamics engine (22) have made it popular with both biologists and physicists. Exploiting these capabilities, a recent work used Cytosim to quantify the macro rigidity modulus specific to organized cytoskeletal structures (e.g., asters). (27). However, rheology and specifically the viscoelasticity of disordered networks have not yet been addressed with Cytosim. Furthermore, a systematic quantification using Cytosim of both microrheology and macrorheology is still missing, leaving a gap in our understanding of how microscopic interactions driven by transient cross-linking, motor activity, and filament turnover translate into emergent mechanical properties in realistic, dynamic, semiflexible filament networks.

**Figure 1:**
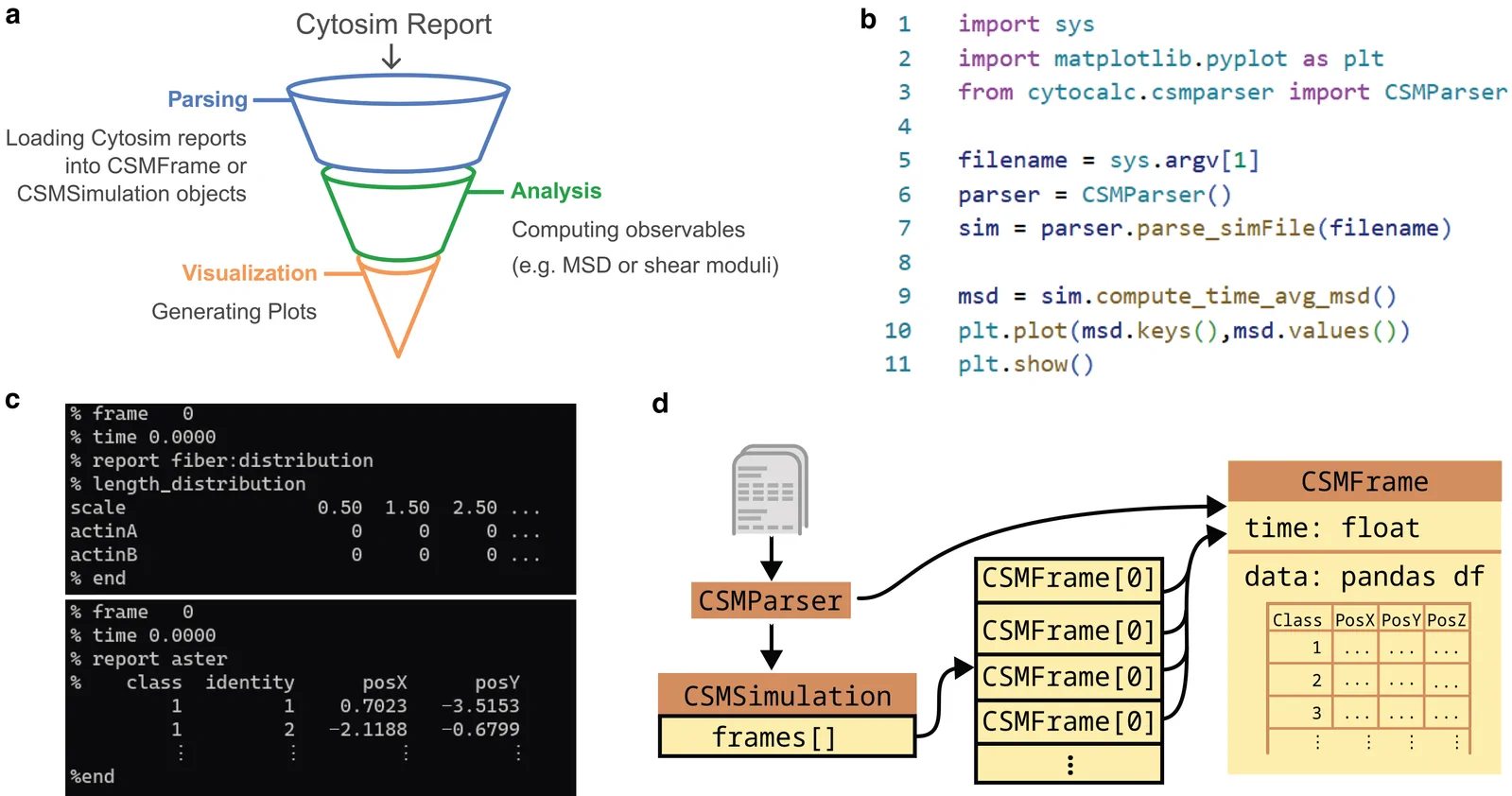
Cytocalc software architecture and workflow. (*a*) Conceptual three-layer design showing the parser, analysis, and visualization layers. (*b*) Example minimal script demonstrating the typical workflow from file parsing to MSD analysis and plotting. (*c*) Sample Cytosim report files illustrating structural differences across output types (here showing data in rows versus columns and different comments) that the parser handles automatically. (*d*) Core module architecture showing the relationship between CSMParser, CSMFrame, and CSMSimulation objects in the data processing pipeline.

Furthermore, the analysis pipeline surrounding Cytosim remains fragmented. Users typically rely on ad hoc scripts to parse Cytosim’s plain-text output files and compute quantities such as forces, stresses, or network topology (23) (with the exception of the discontinued Cytoslysis package(28)). The problem is further compounded by the variety of output file types of Cytosim, each with potentially different data formats (see supporting material S1 and an example in Fig. 1 *c*). This lack of standardization complicates postprocessing, limits reproducibility, and creates barriers for new users. A unified, well-tested analysis package would eliminate these bottlenecks, streamline Cytosim adoption, and allow researchers to focus on scientific questions rather than data wrangling.

To support both routine and advanced analyses, such a package must accommodate the diverse scientific questions posed by Cytosim users. Biologists may be interested in metrics such as contraction speed (24,29), network turnover (30), or spatial patterning (31), whereas physicists often seek to quantify stress relaxation (32,33,34), defect dynamics (35), or frequency-dependent shear moduli (10,12,16). Despite this diversity, many core observables such as filament spatial distribution, mean-square displacement (MSD), and filament-motor interactions are shared across studies (24,36). Standardizing how these quantities are extracted and analyzed would enable meaningful comparisons between different simulation setups, reduce duplication of effort, and promote reproducible practices. It would also create a common foundation for more specialized extensions tailored to specific research goals.

Here we introduce Cytocalc (37), a lightweight Python package that provides a unified and extensible analysis pipeline for Cytosim simulations. Cytocalc provides support for parsing standard Cytosim output types (termed reports), computing some commonly used observables, and generating basic visualizations suitable for downstream analysis or publication. Cytocalc is an open-source package designed for use in automated analysis workflows and can be extended with new features by contributing users. All code, documentation, and simulation files are freely available (37) to support reproducibility and community use.

Crucially, Cytocalc implements, for the first time to our knowledge, a workflow to systematically measure rheological properties directly from Cytosim simulations. After outlining the implementation and capabilities of Cytocalc and validating its output against published results, we apply it to characterize the rheological properties of various cross-linked actin networks. Using Cytosim to study the rheology of cytoskeletal systems allows to link well-studied macroscopic rheological properties to the molecular properties of their constituents and to the composition of the network. Here, we focus on the cross-linker density. We first determine the storage and loss moduli by tracking individual filament segments and the crossover with frequency from predominantly elastic to predominantly viscous behavior, for different solvent viscosities. Then, we determine the dependence of the storage modulus on cross-linker density and show that it exhibits two regimes. We find that for relatively low numbers of cross-linkers, the storage modulus scales with numbers of cross-linkers to the third power, indicative of repression of the thermal bending fluctuations of a purely entangled network as the main source of elasticity in agreement with earlier theoretical predictions (17,38). At higher cross-linker numbers the behavior transitions to a possible power law with an exponent of a half. These results together with the computational workflow proposed here provide a tool for further investigation of cytoskeletal rheology.

## Materials and methods

### Core architecture of Cytocalc

Cytocalc follows a simple three-layer conceptual design (Fig. 1): a dedicated parser to read Cytosim report files, an analysis layer, and a lightweight visualization layer. Clear, well-documented interfaces between these layers allow users to extend or replace components without touching the core logic. The parser supports most standard Cytosim output files (see supporting material S1) and is implemented in the CSMParser module, which handles differences in file structure across report types (see, e.g., Fig. 1 *d* and *e*). Data from a single simulation time frame are stored in a CSMFrame object, whereas a time series of such CSMFrame objects can be collected in a CSMSimulation object. These serve as the main entry point for further analysis, where users can access individual frames or call built-in methods to compute time series or time-averaged observables directly from the CSMSimulation object.

### Extensible analysis layer

The analysis layer provides functions to compute a growing set of observables from the simulation data. The layer includes functions to calculate the spatial distribution of cytoskeleton components and its time evolution, as well as microrheology methods as expanded on in a separate subsection below. Further functions for analysis of nondynamic filament networks can be easily incorporated from external sources such as freud (39). A key quantity characterizing filament segment dynamics and the basis of the microrheology measurements described below is the MSD. Currently, MSD calculations support both displacement-from-origin and time-averaged ensemble methods, with optional correction for periodic boundaries.

### Visualization layer for processed data

Cytocalc provides a small set of wrappers built on Matplotlib to simplify the visualization of key observables such as contraction radius, MSD curves, and viscoelastic spectra. These functions offer sensible defaults for layout and scaling, making them useful for quick inspection or figure preparation. Although more advanced or customized plotting is left to the user, saved figures include metadata recording the Cytocalc version used to generate them, supporting reproducibility and basic provenance tracking.

### Community-driven development and maintenance

To facilitate contributions by different research groups and establish Cytocalc as a community-driven tool, the software is released as open source under the MIT license (37), with the complete source code, scripts, and documentation hosted on GitLab. Contributors can add new analysis routines by extending existing classes or introducing standalone modules without modifying the core parser or simulation logic. Cytocalc is written in Python and builds on the standard scientific-Python stack: NumPy, pandas, SciPy, and Matplotlib, interfacing with the freud analysis library (39), to balance ease of installation with computational efficiency. Comprehensive unit tests cover file parsing, object construction, and analysis routines, with automated testing through GitLab Continuous Integration to help maintain stability across future changes. Performance evaluations demonstrate reliable handling of large Cytosim output files ranging from a few to several hundred megabytes under typical desktop conditions (supporting material S1).

### Microrheology quantification workflow

The complex shear modulus, $G^{*}\left(\omega \right)$, characterizes the rheological response of a material to oscillatory shear perturbations of frequency ω and infinitesimal amplitude (linear rheology). The real part, $G^{\prime }$ (storage modulus), measures the in-phase, elastic-like response, whereas the imaginary part, $G^{\prime \prime }$ (loss modulus), measures the out-of-phase, viscous-like response. Analogously, it is possible to characterize the material response to an applied infinitesimal shear stress, i.e., its deformation compliance, by introducing the complex creep compliance $J^{*}\left(\omega \right)=1/G^{*}\left(\omega \right)$.

Cytocalc implements two complementary methods to compute $G^{*}$, and both are employed in this study. These methods rely on the generalized Stokes-Einstein relations (GSER), which relate $G^{*}$ to the MSD of a probe object in equilibrium with a continuum bath, under the assumption that the surrounding flow field is well described by Stokes flow on the relevant scales. Further details on the derivation, assumptions, and limitations of this approach are provided in supporting material S3. These methods have also been applied in cell environments, where they supply insightful though incomplete information (40,41). The first method, introduced by Mason et al. (42), relies on an algebraic expansion of the MSD on logarithmic timescales around the studied frequency. This yields an analytical approximation of the GSER, which is accurate for smooth MSD time dependences. The second method, proposed by Evans et al. (43), is more computationally involved but offers a more robust approach valid for more realistic viscoelastic fluids. By estimating $G^{*}$ from discrete time measurements of the creep compliance, it avoids artifacts that can result from Fourier transforms, interpolations, and expansions (see supporting material S3).

## Results

### Validation: Actin-myosin network contraction

**Figure 2:**
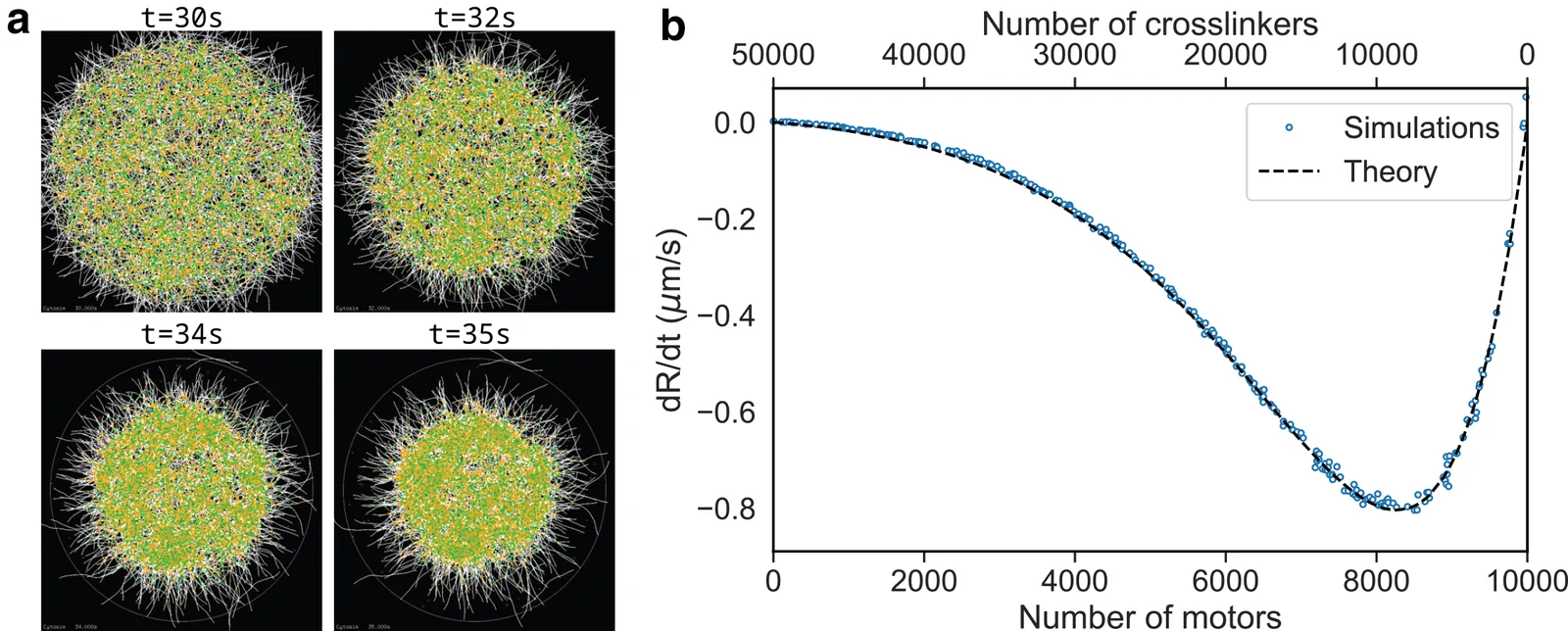
Validation for acto-myosin network contraction: reproducing results of Ref. (24). (*a*) time series of snapshots of a contracting 2D network (7500 motors) as in Ref. (24). (*b*) Rate of change of network radius (R) versus number of cross-linkers and number of motors, in good agreement with the theoretical curve from Ref. (24).

To ensure the reliability of Cytocalc’s output, we first validated its ability to reproduce known dynamic observables from established Cytosim simulations. As a test case, we considered a published model of actomyosin contraction by Belmonte et al. (24), in which a minimal setup of semiflexible filaments, motors, and cross-linkers generates steady contraction over time (see snapshots from a reproduction of that work in Fig. 2 *a*). Cytocalc computes the radius of the actin network at each time point by extracting filament coordinates from the frame report and evaluating their spatial distribution. The contraction rate is then obtained by a linear fit of the network radius versus time (see details in supporting material S2). The resulting contraction profile agrees well with the published results as shown in Fig. 2 *b*, where we plot our simulation results together with the theoretical result from Ref. (24) (which also showed good agreement with previous simulations), indicating that Cytocalc reliably extracts dynamic structural properties from Cytosim simulations.

### Quantification of viscoelasticity of cross-linked actin networks

**Figure 3:**
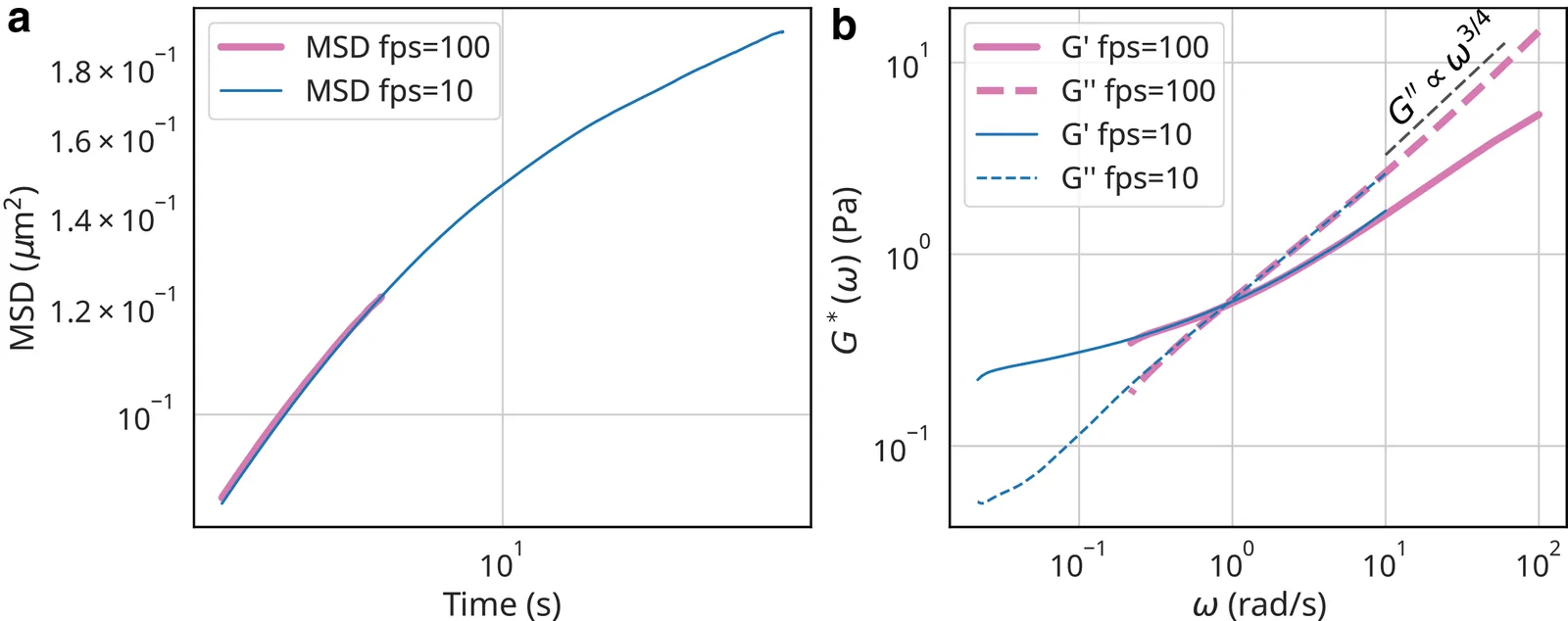
Quantification of frequency-dependent shear modulus from Cytosim data. (*a*) the MSD of filaments over time. (*b*) the storage and loss moduli calculated from the MSD using Mason’s method (42). The data points come from two groups of simulations: one with a (postequilibration) simulation time of 50 s and a frame-saving rate (fps: frames per second) of 10 Hz (*blue*) and the other with a simulation time of 5 s and a frame-saving rate of 100 Hz (*pink*). This allows us to sample a wider frequency range without increasing data size. The cubic simulation box volume is 21.95 $\mu m^{3}$, total length of filaments is 750 μm, numbers of cross-linkers 1600, and solvent viscosity 0.1 Pa·s. See supporting material S4 for the remaining parameters.

To evaluate Cytocalc’s reliability also in a soft-matter physics context, where Cytosim has not been utilized so far, we turned to the viscoelastic properties of cross-linked filament networks. These have been previously studied with particle-based simulations (to our knowledge not open source) by Kim et al. (16). We simulated a dense, 3D, permanently cross-linked, actin-like system (without motors) in Cytosim with 1600 cross-linkers and a solvent viscosity of 0.1 Pa·s. The remaining parameters were adapted to those used in the study by Kim et al. (see supporting material S4), so the comparison with that study provides additional validation of Cytocalc. We tracked the motion of filament segments over time. Cytocalc computes the MSD of these segments using a time-averaged ensemble method (Fig. 3 *a*) and infers the frequency-dependent complex shear modulus $G^{*}\left(\omega \right)$ (Fig. 3 *b*).

We computed the MSD of the filament segments (Fig. 3 *a*) and the corresponding real and imaginary component of the complex shear modulus $G^{*}\left(\omega \right)$ using the method by Mason et al. (42) following the same procedure as Kim et al. (16) (Fig. 3 *b*). This agrees well with the $G^{*}\left(\omega \right)$ computed using the method by Evans et al. (43); see Fig. S2. To extend the accessible frequency range without increasing the total simulation time by orders of magnitude, we analyzed the same simulation trajectory using two different output frame rates. Specifically, we used data saved at 100 frames per second (pink symbols) and at 10 frames per second (blue symbols). The resulting curves show excellent agreement with each other in the overlapping frequency range, indicating that Cytocalc’s rheology output is robust to the choice of sampling interval. Small deviations near the frequency bounds are expected due to resolution limits. Compared with the study by Kim et al. (16), our approach extends the measurable spectrum toward lower frequencies. This allows us to observe the intersection point of $G^{\prime }$ and $G^{\prime \prime }$, below which elasticity dominates, and crossing over to a viscosity dominated regime at higher frequencies, a feature not clearly resolved in the data of Kim et al.

$G^{\prime \prime }$ shows a scaling $∼\omega^{3/4}$ for frequencies above the crossover point. This can be rationalized by simple scaling arguments of the typical bending mode relaxation following the derivations in Refs. (12) and (44) (see supporting material S5), suggesting that cross-linkers hinder the thermal bending of filaments. A further investigation of $G^{*}$ at higher frequencies might be required to confirm this reasoning, which also predicts a scaling of the storage modulus $G^{\prime }∼\omega^{3/4}$.

**Figure 4:**
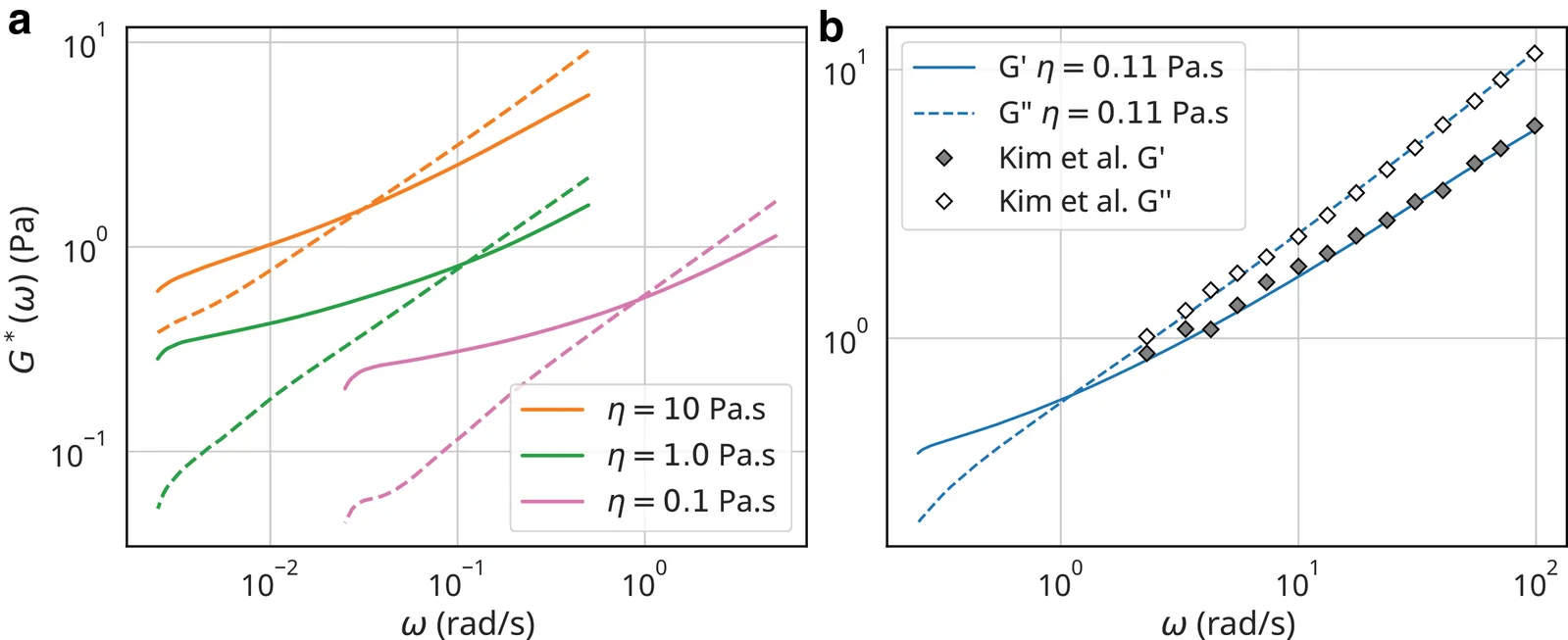
Viscosity dependence of the frequency-dependent shear modulus. (*a*) The real (*lines*) and imaginary (*dashed lines*) components of the frequency-dependent shear moduli for different values of medium viscosity: 10 Pa·s (*orange*), 1 Pa·s (*green*), and 0.1 Pa·s (*pink*). All other parameters are as in Fig. 3. (*b*) Comparison of the frequency-dependent complex shear modulus for medium viscosity η=0.11 Pa·s with the storage and loss moduli reported by Kim et al. (16) (data were extracted using Engauge Digitizer (45)).

Going beyond the previous study (16), we examined how the shape of $G^{*}\left(\omega \right)$ depends on the solvent viscosity (shown by the different colors in Fig. 4 *a*). We observe that this parameter influences both the scale of the moduli and their frequency dependence. Increasing the viscosity shifts the crossover from predominantly elastic to predominantly viscous behavior toward smaller frequencies, whereas both moduli increase. As the solvent viscosity was not explicitly specified in the study of Kim et al. (16), we use it as an effective fitting parameter to align our results with those of their study. The result is shown in Fig. 4 *b*: we directly compare there our results to those of Kim et al. (16), setting the solvent viscosity to 110 times that of water, $\eta =110\;\eta_{w}=0.11$ Pa·s. With this choice, good agreement is obtained, further validating our approach. These results demonstrate that Cytocalc can be used to calculate microrheological properties of cytoskeletal systems, even extending the accessible dynamic range compared with previous simulations.

### Cross-linker dependence of viscoelasticity

Finally, we use rheology measurements implemented in Cytocalc to study how cross-linker density affects the viscoelastic response of passive actin networks. We simulated motor-free cross-linked networks in Cytosim, systematically varying the numbers of cross-linkers (supporting material S4). All other parameters remained unchanged. For each network, we inferred the complex shear modulus $G^{*}\left(\omega \right)$ from the MSD. Here, we employed the method proposed by Evans et al. (43) to avoid low-frequency artifacts in $G^{\prime }$ in systems with higher cross-linker counts (supporting material S3.A).

**Figure 5:**
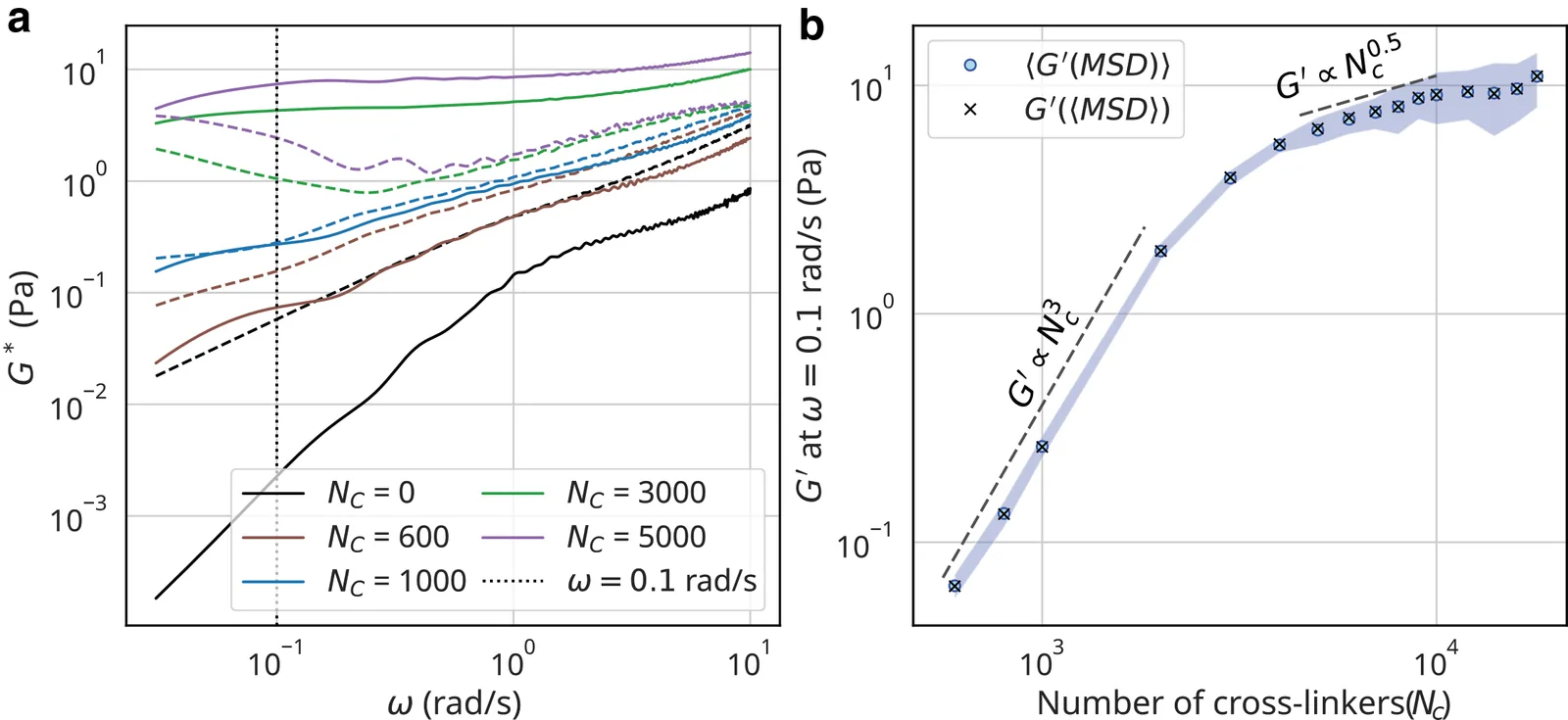
Frequency-dependent shear modulus as a function of cross-linker density/number of cross-linkers. (*a*) The real (*solid lines*) and imaginary (*dashed lines*) components of the frequency-dependent shear moduli, computed using the method proposed by Evans et al. (43) from MSD for different numbers of cross-linkers (*shown by the color*). (*b*) Elastic modulus at ω=0.1 Hz against number of cross-linkers. The shaded region indicates the standard deviation of $G_{0.1}^{\prime }$ computed for each trajectory. Solvent viscosity 0.1 Pa·s and all other parameters as in Figs. 3 and 4.

The storage modulus $G^{\prime }\left(\omega \right)$ (lines) and loss modulus $G^{\prime \prime }\left(\omega \right)$ (dashed lines) are shown for representative cross-linker numbers in Fig. 5 *a* as a function of frequency for representative numbers of cross-linkers, denoted by different colors (data for the full range of cross-linker numbers analyzed appear in Fig. S4). As the number of cross-linkers increases, both $G^{\prime }$ and $G^{\prime \prime }$ increase across all frequencies, reflecting enhanced stiffness and dissipation. The increase of the storage modulus is, however, more pronounced than the increase in the loss modulus: without cross-linkers, the network is mainly viscous as expected, as expressed by a loss modulus much greater than the storage modulus. With more cross-linkers, the entropy reduction due to entanglement constraints leads to a relative increase in the elastic response, and a crossover appears within our frequency range, similar to Figs. 4 and S4. As cross-linker numbers increase even further, the storage modulus surpasses the loss modulus in the entire frequency range we can access, and the material response is effectively elastic.

To assess the effect of permanent cross-linkers on the elasticity of the network, we study the scaling of $G^{\prime }$ with the number of cross-linkers $N_{c}$ in the limit of small frequencies as shown in Fig. 5 *b*. In practice, we determine $G^{\prime }$ for a fixed, small, but finite frequency $\omega_{0}=0.1\;Hz$, and we define $G_{0.1}^{\prime }=G^{\prime }\left(\omega =0.1\right)$. This measure provides a practical proxy for the long-time plateau of the elastic modulus following the approach employed in Refs. (17,38). The average of $G_{0.1}^{\prime }$ calculated from the MSD average of all realizations (crosses) is consistent with the average taken over the $G^{\prime }$ extracted from the MSD of each realization separately (blue circles, with the shaded region indicating the standard deviation), as expected from an equilibrium system.

We observe two distinct regimes in Fig. 5 *b*: increasing $N_{c}$, we find a cubic scaling region $G_{0.1}^{\prime }∼N_{c}^{3}$, eventually crossing over to an approximate scaling $∼N_{c}^{0.5}$ for high cross-linker number. The first region suggests that the low-frequency elastic response is dominated by entropic bending fluctuations of filament segments between adjacent cross-links, as one can understand as follows: if the cross-linkers are approximately uniformly distributed, the average distance between them along a filament is $L_{c}∼N_{c}^{-1}$. Each filament segment of length $L_{c}$ behaves as an entropic spring, with stiffness set by the suppression of thermal bending modes leading to a prediction of $G_{0.1}^{\prime }∼L_{c}^{-3}∼N_{c}^{3}$, in excellent agreement with our data and consistent with theoretical predictions for affine cross-linked networks of semiflexible polymers (17,38).

At higher cross-linker densities, however, the dependence of $G_{0.1}^{\prime }$ on $N_{C}$ weakens, approaching an effective exponent close to 0.5 for $N_{C}$ up to $∼10^{4}$. Above this point the increase of $G_{0.1}^{\prime }$ further reduces, which agrees with our expectation of the finite number of cross-linker binding sites on the filaments getting fully occupied at $N_{c}∼10^{4}$ (see supporting material S4). The deviation from the simple affine thermal model suggests that additional mechanisms become important once the network is densely cross-linked. Possible contributing factors include the suppression of nonaffine bending modes, changes in network architecture (46), or the onset of stretching-dominated elasticity (47).

Overall, our results align with current understanding of the mechanics of cross-linked semiflexible polymer networks and demonstrate how Cytocalc can be used to probe scaling behavior in cytoskeletal assemblies.

## Discussion

In this work, we introduce Cytocalc, an open-source Python package for analyzing Cytosim simulations. Although broadly applicable to a range of problems in the biology and physics of the cytoskeleton, leveraging the versatility of Cytosim as a simulation platform (22), Cytocalc’s initial functionalities are particularly well suited for investigating the rheology of cytoskeleton networks. We validate the software against established results in cytoskeletal mechanics, including contraction dynamics (24) and linear viscoelasticity (16).

Using Cytocalc, we then determine the frequency-dependent shear modulus of cross-linked semiflexible filaments. The calculated storage and loss moduli agree with previous studies (16) and extend the accessible frequency range. This extended range reveals the characteristic crossover frequency (depending on medium viscosity), where the network transitions from predominantly elastic to predominantly viscous behavior. We observe that for relatively low concentrations of cross-linkers (but still high enough not to constitute a purely entangled melt), the high-frequency shear modulus is proportional to ${\left(i\omega \right)}^{3/4}$, suggesting an affine response of the cross-linked network (17). We then systematically vary the number of cross-linkers to study how the low-frequency elastic modulus varies with the cross-linker density and observe a crossover from a thermal affine response $\left(G_{0.1}^{\prime }∼N_{c}^{3}\right)$ to an approximate scaling $∼N_{c}^{0.5}$, whose physical origin remains an open question for future work. Indeed, the integration of Cytosim with the Cytocalc analysis pipeline now enables a streamlined workflow for future studies of cytoskeleton rheology, including for composite networks and uniquely active processes such as dynamic cross-linkers, filament polymerization, and turnover.

Cytocalc fills a gap in the Cytosim analysis pipeline by providing a transparent and extensible framework for computing dynamical, structural, and mechanical observables from raw simulation output. Its modular design—separating parsing, analysis, and visualization—enables reproducible workflows and lowers the barrier to systematic postprocessing. We note a partial functionality overlap with PyCytosim (48), a C-Python interface developed by Serge Dmitrieff that operates on Cytosim source code directly and thus provides comprehensive access to all native Cytosim functions and objects. This offers greater functionality but requires more technical expertise and is less readily integrated into existing research workflows. The no longer maintained Python package Cytolysis (28) is structurally similar to Cytocalc, but it focuses primarily on parsing the report files and does not contain advanced analysis functionalities like the ones presented here for quantifying viscoelasticity.

Although the current Cytocalc version supports a limited set of observables, the codebase is designed for expansion. Additional metrics—such as filament curvature, orientation correlations, or turnover rates—can be incorporated without modifying the parser or core data structures. Current development focuses on both structural improvements, such as a clearer class separation for parsing and analysis (49), and on expanding analysis functionality to incorporate further independent rheological estimators such as macrorheology and two-point microrheology (50,51). Possible challenges for such future works could stem from the inherent limitation of the available frequency range, which is restricted by available computational time and by short enough time steps that prohibit filament crossing. Further limitations could manifest in systems with dynamic turnover where tracking filament segments is more challenging, since segments can vanish and whole filaments can dissolve. Moreover, the predicted viscoelastic spectrum has to be validated, since the GSER may not be satisfied for different systems, as discussed in supporting material S3. The independent rheological estimators would enable cross-validation of the viscoelastic spectrum.

Looking forward, we envision community-driven development of specialized modules, as well as integration with experimental data streams or other simulation platforms. Indeed, as a modern open-source tool built on standard Python libraries, Cytocalc provides a foundation for building robust, interoperable workflows in cytoskeletal simulation research.

## Data and code availability

The Cytocalc source code can be found on GitLab (37). The Cytosim configuration files, report files, and analysis scripts corresponding to each file have been deposited at GRO.data: JGDI8Y (52), and they are publicly available as of the article publication date.

## Acknowledgments

We would like to thank Francois Nedelec, Serge Dmitrieff, Kim Taeyoon, Anastasiia Smagliuk, and Denis Wittig for discussions, feedback, and suggestions. We acknowledge support from the 10.13039/501100001659Deutsche Forschungsgemeinschaft (DFG, German Research Foundation) – project-ID 449750155 – RTG 2756, projects A3 (to K.I.V.S., K.B., and S.K. and Y.G.P.) and A5 (to R.M. and P.K.S.). Simulations were run on the GoeGrid cluster at the University of Göttingen, which is supported by the 10.13039/501100001659Deutsche Forschungsgemeinschaft (project IDs 436382789 and 493420525) and MWK Niedersachsen (grant no. 45-10-19-F-02).

## Author contributions

Y.G.P. designed the research. K.I.V.S., K.B., and R.M. performed the research. K.I.V.S. carried out simulations, built the software, and performed visualization. K.I.V.S. and R.M. analyzed the data. R.M., P.K.S., and S.K. provided the theoretical framework and interpretation of results. K.I.V.S., K.B., R.M., P.K.S., S.K., and Y.G.P. wrote the manuscript. Y.G.P. and K.B. supervised the project.

## Declaration of interests

The authors declare no competing interests.

## Supporting citations

References (53,54,55,56,57,58,59) appear in the supporting material.

## Supporting material

Document S1. Figures S1–S4 and Tables S1 and S2

Document S2. Article plus supporting material

## References

[1] Pollard T.D., Borisy G.G. (2003). Cellular motility driven by assembly and disassembly of actin filaments. Cell.

[2] Rottner K., Stradal T.E.B. (2011). Actin dynamics and turnover in cell motility. Curr. Opin. Cell Biol..

[3] Bray D. (2000). https://www.taylorfrancis.com/books/9781136844355.

[4] Lorenz C., Köster S. (2022). Multiscale architecture: mechanics of composite cytoskeletal networks. Biophys. Rev..

[5] Hohmann T., Dehghani F. (2019). The cytoskeleton—a complex interacting meshwork. Cells.

[6] Michelot A., Berro J., Blanchoin L. (2007). Actin-filament stochastic dynamics mediated by ADF/Cofilin. Curr. Biol..

[7] Zwetsloot A.J., Tut G., Straube A. (2018). Measuring microtubule dynamics. Essays Biochem..

[8] Rigato A., Miyagi A., Rico F. (2017). High-frequency microrheology reveals cytoskeleton dynamics in living cells. Nat. Phys..

[9] Fabry B., Maksym G.N., Fredberg J.J. (2001). Scaling the microrheology of living cells. Phys. Rev. Lett..

[10] Hang J.-T., Xu G.-K., Gao H. (2022). Frequency-dependent transition in power-law rheological behavior of living cells. Sci. Adv..

[11] Kollmannsberger P., Fabry B. (2011). Linear and nonlinear rheology of living cells. Annu. Rev. Mater. Res..

[12] Broedersz C.P., MacKintosh F.C. (2014). Modeling semiflexible polymer networks. Rev. Mod. Phys..

[13] Broedersz C.P., Mao X., MacKintosh F.C. (2011). Criticality and isostaticity in fibre networks. Nat. Phys..

[14] Maxian O., Mogilner A., Donev A. (2021). Integral-based spectral method for inextensible slender fibers in Stokes flow. Phys. Rev. Fluids.

[15] Maxian O., Peláez R.P., Donev A. (2021). Simulations of dynamically cross-linked actin networks: Morphology, rheology, and hydrodynamic interactions. PLoS Comput. Biol..

[16] Kim T., Hwang W., Kamm R.D. (2009). Computational analysis of viscoelastic properties of crosslinked actin networks. PLoS Comput. Biol..

[17] Gittes F., MacKintosh F.C. (1998). Dynamic shear modulus of a semiflexible polymer network. Phys. Rev. E.

[18] Sorichetti V., Lenz M. (2023). Transverse fluctuations control the assembly of semiflexible filaments. Phys. Rev. Lett..

[19] Gong B., Wei X., Lin Y. (2019). Modeling and simulations of the dynamic behaviors of actin-based cytoskeletal networks. ACS Biomater. Sci. Eng..

[20] Liverpool T.B., Marchetti M.C. (2006). Rheology of active filament solutions. Phys. Rev. Lett..

[21] Sheinman M., Broedersz C.P., MacKintosh F.C. (2012). Actively stressed marginal networks. Phys. Rev. Lett..

[22] Nedelec F., Foethke D. (2007). Collective Langevin dynamics of flexible cytoskeletal fibers. New J. Phys..

[23] Lugo C.A., Saikia E., Nedelec F. (2023). A typical workflow to simulate cytoskeletal systems. J. Vis. Exp..

[24] Belmonte J.M., Leptin M., Nédélec F. (2017). A theory that predicts behaviors of disordered cytoskeletal networks. Mol. Syst. Biol..

[25] Letort G., Nedelec F., Théry M. (2016). Centrosome centering and decentering by microtubule network rearrangement. Mol. Biol. Cell.

[26] Akamatsu M., Vasan R., Drubin D.G. (2020). Principles of self-organization and load adaptation by the actin cytoskeleton during clathrin-mediated endocytosis. eLife.

[27] Berg B., Allard J. (2025). Astral architecture can enhance mechanical strength of cytoskeletal networks by modulating percolation thresholds. bioRxiv.

[28] Srj/Cytosim_analysis. GitLab. https://gitlab.com/SergeDmi/cytosim_analysis.

[29] Wollrab V., Belmonte J.M., Koenderink G.H. (2018). Polarity sorting drives remodeling of actin-myosin networks. J. Cell Sci..

[30] Goode B.L., Eskin J., Shekhar S. (2023). Mechanisms of actin disassembly and turnover. J. Cell Biol..

[31] Nédélec F.J., Surrey T., Leibler S. (1997). Self-organization of microtubules and motors. Nature.

[32] Freedman S.L., Banerjee S., Dinner A.R. (2017). A Versatile framework for simulating the dynamic mechanical structure of cytoskeletal networks. Biophys. J..

[33] Mulla Y., MacKintosh F.C., Koenderink G.H. (2019). Origin of slow stress relaxation in the cytoskeleton. Phys. Rev. Lett..

[34] Lieleg O., Kayser J., Bausch A.R. (2011). Slow dynamics and internal stress relaxation in bundled cytoskeletal networks. Nat. Mater..

[35] Mirza W., De Corato M., Arroyo M. (2025). Theory of active self-organization of dense nematic structures in the actin cytoskeleton. eLife.

[36] Ennomani H., Letort G., Blanchoin L. (2016). Architecture and connectivity govern actin network contractility. Curr. Biol..

[37] Iyer K.V.S., Bhattacharyya K., Pollack Y.G. (2025). Cytocalc GitLab repository. https://hdl.handle.net/21.11101/0000-0007-FE56-B.

[38] MacKintosh F.C., Käs J., Janmey P.A. (1995). Elasticity of semiflexible biopolymer networks. Phys. Rev. Lett..

[39] Ramasubramani V., Dice B.D., Glotzer S.C. (2020). freud: a software suite for high throughput analysis of particle simulation data. Comput. Phys. Commun..

[40] Gal N., Lechtman-Goldstein D., Weihs D. (2013). Particle tracking in living cells: a review of the mean square displacement method and beyond. Rheol. Acta.

[41] Soares e Silva M., Stuhrmann B., Koenderink G.H. (2014). Time-resolved microrheology of actively remodeling actomyosin networks. New J. Phys..

[42] Mason T.G. (2000). Estimating the Viscoelastic Moduli of Complex Fluids Using the Generalized Stokes–Einstein Equation. Rheol. Acta.

[43] Evans R.M.L., Tassieri M., Waigh T.A. (2009). Direct conversion of rheological compliance Measurements into Storage and Loss Moduli. Phys. Rev. E.

[44] Morse D.C. (1998). Viscoelasticity of concentrated isotropic solutions of semiflexible polymers. 2. linear Response. Macromolecules.

[45] Mitchell M., Muftakhidinov B., Kylesower (2020). markummitchell/engauge-digitizer: Nonrelease.

[46] Tharmann R., Claessens M.M.A.E., Bausch A.R. (2007). Viscoelasticity of isotropically cross-linked actin networks. Phys. Rev. Lett..

[47] Head D.A., Levine A.J., MacKintosh F.C. (2003). Deformation of cross-linked semiflexible polymer networks. Phys. Rev. Lett..

[48] pycytosim preprint on github. https://github.com/SergeDmi/CytosimPy-article/blob/master/paper.md.

[49] Cytocalc split-analysis branch. https://gitlab.gwdg.de/rtg-2756/Cytocalc/-/tree/split_analysis.

[50] Levine A.J., Lubensky T.C. (2002). Two-point microrheology and the electrostatic analogy. Phys. Rev. E.

[51] Crocker J.C., Valentine M.T., Weitz D.A. (2000). Two-point microrheology of inhomogeneous soft materials. Phys. Rev. Lett..

[52] Iyer V.S.K., Bhattacharyya K., Pollack Y. (2025). Data for “Viscoelasticity analysis of coarse-grained cytoskeletal simulations with Cytosim and Cytocalc”.

[53] Mason T.G., Ganesan K., Kuo S.C. (1997). Particle Tracking Microrheology of Complex Fluids. Phys. Rev. Lett..

[54] Mason T.G., Weitz D.A. (1995). Optical measurements of frequency-dependent linear viscoelastic moduli of complex fluids. Phys. Rev. Lett..

[55] Oppenheim A.V., Schafer R.W., Buck J.R. (1999).

[56] Tassieri M., Evans R.M.L., Cooper J.M. (2012). Microrheology with optical tweezers: data analysis. New J. Phys..

[57] Nédélec F.J. (2022). Cytosim GitLab repository: Polymer melt test. https://gitlab.com/f-nedelec/cytosim/-/blob/main/cym/entangled.cym.

[58] Morse D.C. (1998). Viscoelasticity of concentrated isotropic solutions of semiflexible polymers. 1. Model and Stress Tensor. Macromolecules.

[59] Farge E., Maggs A.C. (1993). Dynamic scattering from semiflexible polymers. Macromolecules.

