# Supplementary material for: Viscoelasticity analysis of coarse-grained cytoskeletal simulations with Cytosim and Cytocalc
Source: Biophys J. 2026 Mar 4;125(7):1614–22. doi: 10.1016/j.bpj.2026.02.026 (PMC13351658; doi:10.1016/j.bpj.2026.02.026)
Supplement: Document S1. Figures S1–S4 and Tables S1 and S2 [file mmc1.pdf]

Biophysical Journal, Volume *125*

Supplemental information

Viscoelasticity analysis of coarse-grained cytoskeletal simulations with Cytosim and Cytocalc

Krishna Iyer V.S., Komal Bhattacharyya, Raffaele Mendozza, Peter K. Sollich, Stefan Klumpp, and Yoav G. Pollack

Supplementary material: Viscoelasticity Analysis of Coarse-grained Cytoskeletal Simulations with Cytosim and Cytocalc

Krishna Iyer V S, Komal Bhattacharyya, Raffaele Mendozza,
Peter K Sollich, Stefan Klumpp, Yoav G Pollack

S1. SUPPORTED REPORT TYPES

As mentioned in Section 2.1, Cytocalc supports the vast majority of Cytosim's different output types. To address the variability in the structure of report files, Cytocalc categorizes them into types I, II or III based on the position of the column headers relative to the line declaring the report type. In type I report files, the header follows the report type, whereas type II files contain a comment between the two lines. Type III files have a header column instead of a header row. Table S1 provides a list of supported report file types, classified into the respective categories. Cytocalc is capable of parsing report files from simulations with any dimension. Technically, positions are stored as an $N \times 3$ array, with missing dimensions defaulting to zero values.

TABLE S1. Supported Report Types

Type I		Type II	Type III
fiber:point	aster	fiber	fiber:distribution
fiber:sample	bead	fiber:confine_force	
fiber:segment	bead:position	fiber:position	
fiber:tension	fiber:age		
network:size	fiber:energy		
organizer	fiber:confinement		
single	fiber:displacement		
single:link	fiber:dynamic		
single:position	fiber:end		
single:state	fiber:force		
solid:hands	fiber:hand		
solid:position	fiber:lattice		
space	fiber:lengths		
sphere:position	fiber:link		
fiber:moment			

In order to test the efficiency of the parser for different file sizes, we simulated systems with 100, 1000, 10000 actin filaments and generated `fiber:point` reports. The time taken by `parse_simFile`, measured on an Intel® Core™ i5-7600 CPU and averaged over 10 attempts, was found to be well under a minute for file sizes as large as 500MB.

TABLE S2. Parser Performance

Filament Count	File Size	Time
100	5.5 MB	3.5 ± 0.1 s
1000	55 MB	5.9 ± 0.8 s
10000	549 MB	27.4 ± 0.9 s

S2. EXTRACTION OF THE CONTRACTION RATE FROM SIMULATIONS

In order to measure the contraction rates discussed in Section 3.1, we simulate a 2D network with motors and cross-linkers using parameters from Belmonte et al.[1]. Following this reference, for each simulation, the network

radius was measured as a function of time:

$$c = \frac{1}{P}\sum_i x_i \tag{S1}$$

$$R = \sqrt{\frac{2}{P}\sum_i |x_i - c|^2} \tag{S2}$$

where c is the center of mass, x_i are the positions of all the filament segments and P is the number of such segments. Eq. S2 provides an estimate for the radius of a disk given P points uniformly distributed on its surface.

In the work of Belmonte et al. [1], the simulation time was short enough to only capture the regime of contraction linear in time, each run simulating only 5 seconds after the initial equilibration. Running the simulation for longer, we observe that the stochastic contraction mechanism exhausts itself, and eventually the decrease in the network radius saturates. We limit ourselves to the initial linear regime for calculating the contraction rate.

To characterize the contraction rate as a function of motor count, we follow the example of Belmonte et al. and run 300 simulations with motor counts chosen from a uniform distribution between 0 and 10000. In each simulation, the cross-linker count is set to $50000 - 5N_m$ where N_m is the number of motors in the system. The results are shown in Fig. 2 and discussed in the main text.

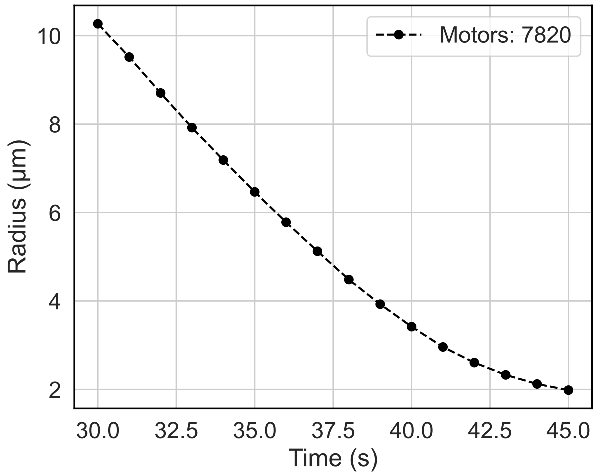

FIG. S1. Network radius vs time for an example simulation with 7820 motors and 10900 cross-linkers. The contraction rate dR/dt is -0.792 μm/s.

S3. METHODS FOR OBTAINING THE SHEAR MODULUS FROM MSD

The mean squared displacement of a particle of radius a in a purely viscous fluid is given by:

$$\langle \Delta r^2(t) \rangle = 6Dt = \frac{k_B T t}{\pi \eta a}. \tag{S3}$$

While the MSD, $\langle \Delta r^2 \rangle$, is typically described as a function of time, we can, in principle, apply a unilateral Fourier transform [2] to Eq. S3 to obtain a frequency dependent mean-squared displacement

$$\langle \widetilde{\Delta r^2} \rangle(\omega) = -\frac{k_B T}{\pi \eta \omega^2 a}, \tag{S4}$$

where $\langle \widetilde{\Delta r^2} \rangle(\omega)$ is the MSD in Fourier space.

The Generalized Stokes-Einstein Relation (GSER) similarly relates the $\langle \widetilde{\Delta r^2} \rangle(\omega)$ of a particle in a non-Newtonian liquid to a frequency dependent viscosity $\eta^*(\omega)$:

$$\langle\widetilde{\Delta r^2}\rangle(\omega) = -\frac{k_B T}{\pi\eta^*(\omega)\omega^2 a}. \tag{S5}$$

From Eq. S5 and Eq. S4, it is evident that the standard Stokes-Einstein relation is retrieved from the GSER for a medium with frequency-independent viscosity η. The relation can be extended to linear viscoelastic media introducing a complex, frequency-dependent viscosity $\eta^*(\omega)$. It is, however, a phenomenological argument that relies on the assumption that the probe particle is in equilibrium with a continuum bath and that the flow field around the particle does not deviate from Stokes law. It is then reasonable to assume that the particle diffusion spectrum will be influenced by the viscoelastic spectrum of the bath [3, 4]. The complex shear modulus can then be calculated from the complex viscosity η^* as:

$$G^*(\omega) = i\omega\eta^*(\omega). \tag{S6}$$

Substituting Eq. S6 into Eq. S5 and rearranging gives explicitly:

$$G^*(\omega) = \frac{k_B T}{i\pi\omega a\langle\widetilde{\Delta r^2}\rangle(\omega)}. \tag{S7}$$

With Eq. S7 one then has a theoretical framework for extracting the complex shear modulus $G^*(\omega)$ from the Fourier transform of the mean squared displacement. The validity of the GSER must, however, be assessed for the system under consideration. Comparisons with macroscopic rheometry have shown excellent agreement with the GSER from Eq. S7, as reported for example in ref. [2, 3]. An exhaustive discussion about the validity of the GSER is beyond the scope of this appendix, but the interested reader can find it in ref. [4].

A. Mason's method

Using the GSER to calculate the shear moduli requires performing a unilateral Fourier transform on an arbitrary MSD obtained from simulation (or indeed experiment). Such numerical integration can often induce hard-to-control errors. The method proposed by Mason et al. in Ref. [2] is based on a linear expansion of $\ln\left(\langle\Delta r^2(t)\rangle\right)$ around $t = 1/\omega$, where ω is the frequency being considered. After performing the Fourier transform one obtains

$$i\omega\langle\widetilde{\Delta r^2}\rangle(\omega) \approx \langle\Delta r^2(1/\omega)\rangle\Gamma\left(1+\alpha(\omega)\right) i^{-\alpha(\omega)}, \tag{S8}$$

where $\Gamma(1+\alpha)$ is the Gamma function and $\alpha(\omega)$ is defined as:

$$\alpha(\omega) = \left[\frac{d\ln\langle\Delta r^2(t)\rangle}{d\ln t}\right]_{t=1/\omega}. \tag{S9}$$

Using this approximation in Eq. S7 one finds the following expressions for the storage (G') and loss (G'') moduli:

$$\begin{aligned} G'(\omega) &= |G^*(\omega)|\cos(\pi\alpha(\omega)/2), \\ G''(\omega) &= |G^*(\omega)|\sin(\pi\alpha(\omega)/2), \end{aligned} \tag{S10}$$

with $|G^*(\omega)|$ given by:

$$|G^*(\omega)| \approx \frac{k_B T}{\pi a\langle\Delta r^2(1/\omega)\rangle\Gamma\left(1+\alpha(\omega)\right)}. \tag{S11}$$

This method intrinsically assumes that the MSD will vary on logarithmic timescales, so it is expected to be more accurate when the MSD is sufficiently smooth. Moreover, the estimate of G' (resp. G'') becomes less accurate for $\alpha \approx 1$ and $\alpha \approx 0$. These correspond, respectively, to regimes of ballistic growth of the MSD and to a t-independent plateau in the MSD.

B. Evans' method

While Eq. S11 is a straightforward technique for obtaining the frequency-dependent shear moduli, the approximate Fourier (or Laplace) transforms may affect the accuracy of the result. An improved algorithm was devised in Ref. [5] that sidesteps this by directly reconstructing a discretized complex shear modulus starting from the creep compliance $J(t)$. The compliance is a measure of 'deformability' of a material, i.e. the amount of shear produced by a unit stress. For elastic solids, it is the inverse of the shear modulus:

$$GJ = 1. \tag{S12}$$

For viscoelastic materials, Eq. S12 generalizes to a convolution in the time domain [5], which in the frequency domain again becomes simply a product:

$$G^*(\omega)J^*(\omega) = 1. \tag{S13}$$

The fourier transform of the creep compliance $J(t)$ is defined as,

$$\tilde{J}(\omega) = \int_{-\infty}^{\infty} J(t)e^{-i\omega t}dt \tag{S14}$$

Combining Eqs. S7 and S13, one sees that MSD data give direct access to compliance estimates via

$$\langle \Delta r^2(t) \rangle = \frac{k_B T}{\pi a} J(t). \tag{S15}$$

It is reasonable to assume that a viscoelastic fluid will have a long-time viscous behavior, resulting in a linear scaling $J \sim t/\eta$, where η is the steady state viscosity of the material, which needs to be extrapolated from the data. As a result, the second time derivative $\ddot{J}(t)$ of $J(t)$ *has* to vanish at long times. Moreover, causality requires that $J(t)$ (and hence $\ddot{J}(t)$) must vanish for $t < 0$ [5]. It follows that the Fourier transform of $\ddot{J}$ converges under all conditions, and can be used to reconstruct the Fourier transform $\tilde{J}(\omega)$ of $J(t)$:

$$\tilde{J}(\omega) = -\frac{1}{\omega^2}\tilde{\ddot{J}}(\omega). \tag{S16}$$

Accounting for the discontinuity at $t = 0$ induced by the time resolution of the measurements, one can then obtain

$$\tilde{\ddot{J}}(\omega) = i\omega J(t = 0) + \dot{J}(t = 0) + \tilde{\ddot{J}}_+, \tag{S17}$$

where J_+ is the creep compliance restricted to strictly positive times.

In practice, we approximate $J(t)$ with a piecewise linear function passing through the measurement points $J_1, \ldots, J_N$ at sampling times $t_1, \ldots, t_N$ and perform a Discrete Time Fourier Transform (DTFT) [6] on $\ddot{J}_+(t)$ (now reduced to a series of delta functions at the measurement times) to find $\tilde{J}(\omega)$ by combining Eqs. S13, S16 and S17. This leads to [5, 7] :

$$\frac{i\omega}{G^*(\omega)} = i\omega J(0) + (1 - e^{-i\omega t_1})\frac{J_1 - J(0)}{t_1} + \frac{e^{-i\omega t_N}}{\eta} + \sum_{k=2}^{N}\left(\frac{J_k - J_{k-1}}{t_k - t_{k-1}}\right)\left(e^{-i\omega t_{k-1}} - e^{-i\omega t_k}\right). \tag{S18}$$

This method is independent of the sampling frequency of the $J(t)$ measurements (which is *not* required to be uniform), but of course this sampling frequency still affects the resolution in frequency space, which is now limited by the *Nyquist–Shannon* theorem. On the other hand, the method is essentially based on the discrete implementation of an integral transform, so measurement noise effects are expected to accumulate. An improved version of the algorithm explicitly dealing with these limitations is provided in Ref. [7].

C. Comparison of Mason's and Evans' methods

Both the methods by Mason and Evans presented above were employed in this work for reasons already explained in the main manuscript. Here we compare the output of these two methods. Fig. S2 shows the output of these two methods for the same simulation data as used in Figure 3 of the main manuscript (1600 cross-linkers). The frequency dependent storage and loss moduli obtained using Mason's method are shown as pink lines, while a similar output of Evan's method is shown in blue lines. The resulting G' and G'' agree well between the two methods, especially for intermediate frequencies.

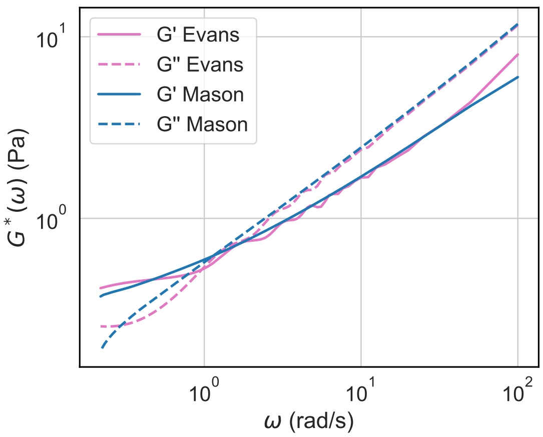

FIG. S2. G' and G'' calculated using Evans' and Mason's methods from the simulation data corresponding to Figure 3 (1600 cross-linkers). The two results are in good agreement. Small discrepancies are apparent for the extrema of the frequency range.

D. Segment Tracking Microrheology

Above, we described the GSER for a spherical probe particle. To analyze our simulations, we in fact employ Segment Tracking Microrheology, a technique devised in Ref. [8], where filament segments are used as the probe instead of beads. This requires adapting Equations Eq. S11 and Eq. S16 for cylindrical probes of diameter σ and length L. This is achieved by replacing the radius of the bead a by an effective radius r_b that satisfies the following relation:

$$\zeta_\perp = 6\pi\eta r_b \,, \tag{S19}$$

where

$$\zeta_\perp = 3\pi\eta\sigma\frac{3 + 2L/\sigma}{5} \,. \tag{S20}$$

S4. RHEOLOGY SIMULATION: CYTOSIM PARAMETERS CORRESPONDING TO KIM ET AL.

In Section 3.2 of the main text, we discuss simulations performed with Cytosim aimed at studying the viscoelastic properties of actin networks. For this purpose, we prepare a cross-linked network of actin-like filaments in Cytosim, adapting parameters from Kim et al.[8]. Table S3 lists the common parameters used in the simulations. A cubic box of side length 2.8 μm with periodic boundary conditions (PBC) is initialized with 500 filaments of length 1.5 μm. The length of each segments of the filaments is 0.07 μm. The total number of cross-linker binding sites is the number of filaments $\times$ filament length/segmentation length $= 10,714$. After equilibration[9] for 5 s, cross-linkers are added, and the system is equilibrated again for 50 s. The equilibration time with cross-linkers is chosen such that all cross-linkers are bound to two filaments, see Fig. S3 (and see Sec. S4 A below for the derivation of the analytical estimate). The timestep used is short enough to ensure that the thermal fluctuations are much smaller than the steric repulsion range of 0.007 μm, which prevents artifacts of filament crossing and topological inconsistencies [10].

The highest meaningful frequency for measurements of $G^*(\omega)$ is determined by the frame-rate[11] and the lowest by the total duration of the simulation. However, when running long simulations at high frame-rates, the amount of data produced increases exponentially with each additional decade in frequency. To avoid this, different simulations are run for different ranges of frequency with a reasonable balance between duration and frame-rate. For example, we use post-equilibration measurement durations of 5 s (high frame-rate), 50 s and 500 s (longer durations), saving 500 regularly spaced frames in each case. With this setup, the data storage requirements grow linearly with the number of decades in frequency.

To obtain ensemble-averaged measurements, we ran 80 'trials' for each system configuration. These were run in batches on the Göttingen University Physics Department's GoeGrid computing cluster. With filament probes, as in the case of Segment Tracking Microrheology, random filament segments (one per filament) were tracked, with the MSD averaged over all probes across all trials.

To quantify the effect of cross-linking, we varied the number of cross-linkers in the system from $N_C = 600$ to $N_C = 18000$. The complex shear modulus was then computed from the MSD for each trial and subsequently averaged

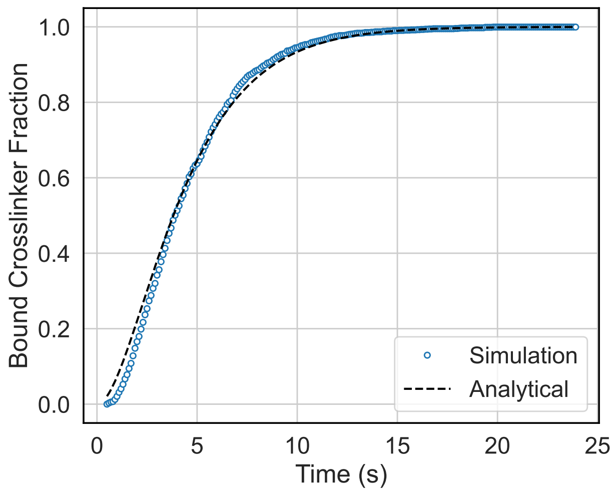

FIG. S3. Fraction of cross-linkers bound to two filaments vs time. Derivation of the analytical expression is given in Sec. S4 A

TABLE S3. Parameters of benchmark simulation. Fil: filament, Cl: cross-linkers

Parameter	Value
Timestep	0.0005 s
Box Length	2.8 μm
Periodicity	xyz
Viscosity	0.1 Pa.s
Fil. length	1.5 μm
Fil. rigidity	0.001 pN/μm^2
Fil. Segmentation	0.07 μm
Fil. Steric Range	0.007 μm
Fil. Steric Force	16.9 pN
Cl. Binding Rate	10 s^{-1}
Cl. Binding Range	0.02 μm
Cl. Unbinding Rate	0 s^{-1}
Cl. Stiffness	4.23 pN/μm
Cl. Diffusion	10 μm^2/s

over all trials. This was found to be consistent with the complex shear modulus obtained from the ensemble-averaged MSD (Fig. 5), while simultaneously providing a measure for the variability in the shear moduli. Fig. S4 shows the curves for G' and G'' for the full data set of different cross-linker counts. Evans' method was employed for this analysis to avoid low-frequency artifacts produced by Mason's method in heavily-cross-linked networks.

A. Estimating cross-linker binding times

We can provide an analytical estimate for the equilibration time by calculating an effective binding rate for the cross-linkers and solving the corresponding Master equation. Each hand of a cross-linker binds at a rate of κ_b (binding rate $= 10\text{s}^{-1}$) when within a distance r_b (binding range $= 0.002\,\mu$m) of a filament. Therefore, the effective binding rate (k) for a cross-linker is given by:

$$k = P_b \kappa_b,$$

where P_b is the probability for a cross-linker to be in the binding range. We can estimate this using the volume fraction occupied by all N_f filaments, treating each as a cylinder with radius r_b and length L_f to get the effective binding rate

$$k = \kappa_b \frac{N_f L_f \pi {r_b}^2}{V} \approx 0.44 \tag{S21}$$

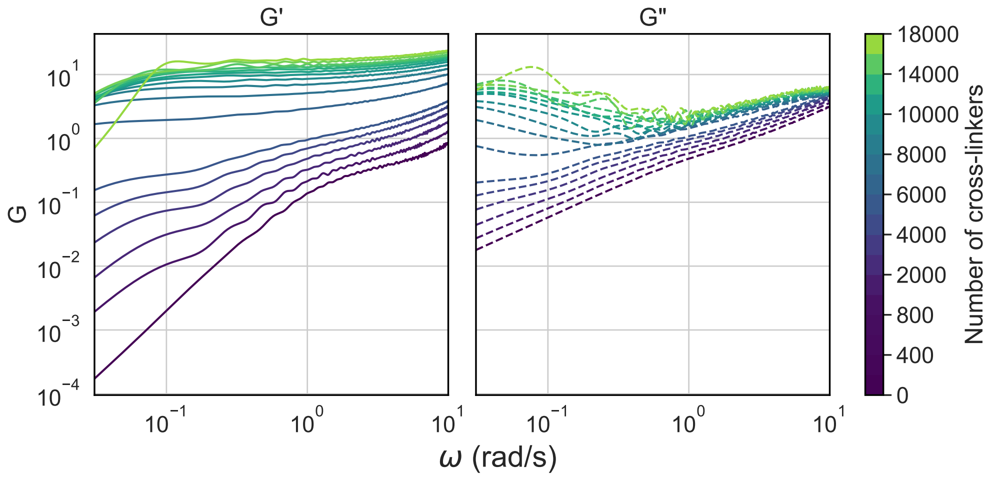

FIG. S4. G' (solid, left) and G'' (dashed, right) for different number of cross-linkers shown by the colorbar

Since the network is nearly isotropic, we can assume that the binding rate is identical for both free and singly-bound cross-linkers. Using this, we can write down the Master equation for this system, where ϕ_f, ϕ_s and ϕ_d are the fractions of free, single-bound and double-bound cross-linkers, respectively:

$$\begin{aligned}\dot{\phi}_f &= -k\phi_f \\ \dot{\phi}_s &= k(\phi_f - \phi_s) \\ \dot{\phi}_d &= k\phi_s.\end{aligned} \tag{S22}$$

Solving this Master equation with an initial condition $\phi_f(0) = 1$ we obtain an analytical expression for the fraction of bound cross-linkers:

$$\phi_d = 1 - \exp(-kt)(1 + kt). \tag{S23}$$

We find that this analytical approximation is in good agreement with the fraction of bound cross-linkers measured during the equilibration phase of the simulation (Fig. S3). Importantly, we note that Eq. S23 is independent of the total number of cross-linkers, permitting the use of a constant equilibration time for simulations with different cross-linker counts.

S5. SCALING ARGUMENT FOR $\omega^{3/4}$ POWER-LAW OF G''

Fig. 3 shows that, for frequencies above the crossing point, the loss modulus of the cross-linked actin network obtained using Mason's method scales as $\omega^{3/4}$. The result can be explained on the basis of the high frequency response of semiflexible, entangled polymer networks, as extensively explained in [12] and [13, 14]. For completeness we provide a brief qualitative picture here, to highlight the physical assumptions underlying the observed scalings.

When strained at high frequency, the network response is dominated by the single polymer's transversal oscillations, while the cross-linkers impede filament tangential sliding. By simple scaling arguments, oscillations must increase with temperature T and with the wavelength λ of the bending mode, while decreasing with the bending modulus κ. Defining u as the transversal displacement field from the straight filament configuration, we then expect typical amplitudes $\langle u^2 \rangle \sim \frac{\lambda^3}{\ell_p}$, where ℓ_p is the persistence length ($\ell_p \sim \kappa/T$). It is well known that transversal fluctuations for a polymer in a viscous solution have a dispersion relation $\omega \sim \lambda^{-4}$ [15], so we expect the typical bending mode relaxing after time t to be of wavelength $\lambda \sim t^{1/4}$. Knowing that the transversal fluctuations are dominated by the longest unconstrained bending mode [12] (of wavelength of the order of the entanglement length ℓ_e in our case), then *typical* end-to-end polymer length fluctuations will scale as $\langle u^2 \rangle \sim t^{3/4}$, implying a shear modulus $\sim (i\omega)^{3/4}$[16, 17]. The derivation of this high frequency scaling thus relies on the assumption that the cross-linked network of semiflexible polymers has $\ell_e < \ell_p$; in the other case, which is not explored numerically in this work, simple fluctuations of the order $\sim t^{1/2}$ are expected, as can be easily seen from the above argument considering the longest unconstrained bending

mode to be of length ℓ_p.

---

[1] J. M. Belmonte, M. Leptin, and F. Nédélec, A theory that predicts behaviors of disordered cytoskeletal networks, Mol. Syst. Biol. **13**, 941 (2017).
[2] T. G. Mason, K. Ganesan, J. H. Van Zanten, D. Wirtz, and S. C. Kuo, Particle tracking microrheology of complex fluids, Phys. Rev. Lett. **79**, 3282 (1997).
[3] T. G. Mason and D. A. Weitz, Optical measurements of frequency-dependent linear viscoelastic moduli of complex fluids, Phys. Rev. Lett. **74**, 1250 (1995).
[4] T. G. Mason, Estimating the viscoelastic moduli of complex fluids using the generalized Stokes–Einstein equation, Rheol. Acta **39**, 371 (2000).
[5] R. M. L. Evans, M. Tassieri, D. Auhl, and T. A. Waigh, Direct conversion of rheological compliance measurements into storage and loss moduli, Phys. Rev. E **80**, 012501 (2009).
[6] A. V. Oppenheim, R. W. Schafer, and J. R. Buck, *Discrete-Time Signal Processing*, 2nd ed. (Prentice Hall, 1999).
[7] M. Tassieri, R. M. L. Evans, R. L. Warren, N. J. Bailey, and J. M. Cooper, Microrheology with optical tweezers: data analysis, New J. Phys. **14**, 115032 (2012).
[8] T. Kim, W. Hwang, H. Lee, and R. D. Kamm, Computational analysis of viscoelastic properties of crosslinked actin networks, Plos. Comput. Biol. **5**, e1000439 (2009).
[9] Filaments are initially placed randomly without any bending. This short equilibration imparts Brownian fluctuations to the segments.
[10] F. J. Nédélec, Cytosim gitlab repository: Polymer melt test (2022).
[11] This is the inverse of the time between two saved frames (t_f), which is often longer than the *timestep*.
[12] C. P. Broedersz and F. C. MacKintosh, Modeling semiflexible polymer networks, Rev. Mod. Phys. **86**, 995 (2014).
[13] D. C. Morse, Viscoelasticity of concentrated isotropic solutions of semiflexible polymers. 1. model and stress tensor, Macromolecules **31**, 7030 (1998), https://doi.org/10.1021/ma9803032.
[14] D. C. Morse, Viscoelasticity of concentrated isotropic solutions of semiflexible polymers. 2. linear response, Macromolecules **31**, 7044 (1998), https://doi.org/10.1021/ma980304u.
[15] E. Farge and A. C. Maggs, Dynamic scattering from semiflexible polymers, Macromolecules **26**, 5041 (1993), https://doi.org/10.1021/ma00071a009.
[16] F. C. MacKintosh, J. Käs, and P. A. Janmey, Elasticity of semiflexible biopolymer networks, Phys. Rev. Lett. **75**, 4425 (1995).
[17] F. Gittes and F. C. MacKintosh, Dynamic shear modulus of a semiflexible polymer network, Phys. Rev. E **58**, R1241 (1998).
